# Evidence on the effectiveness of health literacy interventions in the EU: a systematic review

**DOI:** 10.1186/s12889-018-6331-7

**Published:** 2018-12-29

**Authors:** Boudewijn B. Visscher, Bas Steunenberg, Monique Heijmans, Jolien M. Hofstede, Walter Devillé, Iris van der Heide, Jany Rademakers

**Affiliations:** 1 University of Applied Sciences Utrecht, Heidelberglaan 7, 3584 CS Utrecht, The Netherlands; 2 Nivel (Netherlands institute for health services research), Otterstraat 118, 3513 CR Utrecht, The Netherlands; 3 Julius Global Health, Julius Center for Health Sciences and Primary Care, University Medical Center Utrecht, Utrecht, The Netherlands; 4 Department of Family Medicine, Maastricht University, CAPHRI, (Care and Public Health Research Institute), Maastricht, The Netherlands

**Keywords:** Health literacy, Europe, Interventions, Review

## Abstract

**Background:**

In the last decade, the attention for health literacy has increased in the European Union. This is due to three main reasons. First, reviews have shown that inadequate health literacy is associated with worse health outcomes, higher health care use and expenditure. Second, in all European countries the population is aging and the number of chronically ill people is rising. Improving health literacy in this group can offer greater opportunities to take an active part in society, be independent and improve quality of life. Third, since most research on health literacy has been conducted outside Europe and relatively little is known about the development of health literacy interventions and its effects on outcome measures in European countries. The aim of this systematic review was to assess the evidence on the effectiveness of health literacy interventions in the European Union published between 1995 and 2018.

**Methods:**

Searches have been performed in Medline, PubMed, EMBASE, CINAHL, Cochrane library, PsychINFO, ERIC, Web of Science and SCOPUS for publications on health literacy intervention studies in European Union countries. Studies were included if the research was conducted in one or more Member States of the European Union, the publication described an intervention study, the intervention was aimed at health literacy, the publication described an outcome measure related to health literacy and the publication was written in English, French or German.

**Results:**

A total of 23 studies were included. Three types of interventions were identified; aimed at improving health literacy, tailored to different health literacy levels and aimed at improving health outcomes in general that differentiated in effects for people with different health literacy levels. Most interventions identified in the review focus on the functional level of health literacy or numeracy. The strength of evidence from the European health literacy intervention studies was low and there was a huge heterogeneity in study design, measurement tools and outcomes measured.

**Conclusions:**

Promising interventions were tailored to the needs of patients, addressing functional, interactive and critical skills and use not difficult animated spoken text. Future research should focus on the development and assessment of such interventions and use stronger designs.

**Electronic supplementary material:**

The online version of this article (10.1186/s12889-018-6331-7) contains supplementary material, which is available to authorized users.

## Background

Health literacy is a topic of growing importance in European public health research. In general, health literacy is ‘linked to literacy and encompasses people’s knowledge, motivation and competences to access, understand, appraise, and apply health information in order to make judgments and take decisions in everyday life concerning healthcare, disease prevention and health promotion to maintain or improve quality of life during the life course.’ This is the definition of health literacy as it was developed in the European Health Literacy Project (HLS-EU) [1, 2]. This definition includes the public health perspective on health literacy and can also be specified to an individual approach.

Apart from this one, there are many different definitions and conceptualisations of health literacy [2]. Narrow definitions focus on basic literacy (the ability to read and write), while others also include a wider range of cognitive and psychosocial skills in the definition. Furthermore, definitions differ with respect to the actions, information and resources, objectives, context and time aspects which they do or do not include [2]. Nutbeam et al. [3] distinguishes in the broad definition of health literacy three dimensions of health literacy: functional, interactive and critical literacy. Functional health literacy is the ability to read health information. Sometimes numeracy (the ability to use mathematics in everyday life) is also included in the concept of functional health literacy. Interactive health literacy refers to ‘more advanced cognitive and literacy skills which, together with social skills, can be used to actively participate in everyday situations, extract information and derive meaning from different forms of communication, and apply this to changing circumstances.’ [3] Critical health literacy refers to ‘more advanced cognitive skills which, together with social skills, can be applied to critically analyse information and use this to exert greater control over life events and situations’. [3] Most of existing research on health literacy focusses on functional health literacy.

Especially in the last decade, the attention for health literacy has increased in the European Union (EU). This is due to three main reasons. First, studies mainly from the United States of America (USA) have shown that inadequate health literacy is associated with worse health outcomes, poor preventive care behaviours, higher health care service use and expenditures. In addition these studies showed that health literacy influences the effects social determinants of health have on health status and as such is an important determinant of health inequalities [4–8]. Therefore more attention for health literacy can lead to a substantial return at both the individual and the community level, by improving health and well-being on one hand and reducing unnecessary healthcare visits and costs on the other. Second, in all European countries the population is aging and the number of chronically ill people is rising. Much is expected from this group in terms of self-management. However, adequate health literacy is required to fulfil an active role regarding health and healthcare. Third, most research on health literacy has been conducted outside Europe, in the USA and more recently in Japan, Taiwan and Australia. As a consequence, relatively little is known about the development of health literacy interventions and its effects on outcome measures in European countries. While many of the USA studies on health literacy primarily focus on functional health literacy in the clinical or medical setting, EU studies more often use a broader definition of health literacy, and address issues both inside and outside the clinical setting [9]. Instead of a risk (inadequacy in the context of healthcare), health literacy is also defined as an asset, a means to exert greater control over health and over personal, social and environmental determinants of health [2, 3, 9]. Furthermore, the health and social welfare systems between USA and Europe differ. The USA health systems have a limited government involvement with an important role for the private sector stakeholders (e.g. health care providers and insurers) and most of the payment is on fee for service basis [10]. In Europe there is a stronger government involvement than in the USA. In some countries (e.g. the Netherlands) there is a gatekeeping role for the primary care and paying on capitation basis, and in other countries (e.g. the UK) there is a system of National Health Service which offers (predominantly) free health care services [10, 11]. These fundamental differences between the USA and Europe areas an important reason why the largely USA based body of evidence cannot simply be assumed to also be true in a European setting. In the USA, Sheridan et al. [12] and Berkman et al. [13]. Sheridan et al. [12] and Berkman et al. [13] found interventions that improved participants´ comprehension of health information. Moreover, interventions aimed at self-management that took the level of health literacy of patients into account reduced emergency department visits and hospitalizations and self- and disease-management interventions reduced disease severity. Effects of health literacy interventions on other outcomes were mixed or limited. Most studies in the field of health literacy are correlational, there is a lack of convincing studies that show that health literacy can change as a result of an intervention.

To determine the efficacy of health literacy interventions in the EU context, a similar systematic review as the review conducted by Berkman et al. [13] and Dennis et al. [14] in the USA was undertaken for EU countries, using similar search strategies for optimal comparability of the results. The aim of this systematic review is to assess the evidence on the effectiveness of health literacy interventions in the EU published from 1995 until 2018. This is the first systematic review on health literacy interventions in the EU context. The results of this review will be compared to the results of the review in the USA context.

## Methods

This systematic review was conducted in accordance with the PRISMA (Preferred Reporting Items for Systematic Reviews and Meta-Analyses) guidelines [15]. This research is based on and an update of the work done in Work Package 1 of the HEALIT4EU research project, executed under the EU Health Programme (2008–13) in the framework of contract no. 20146201 with the Consumers, Health and Food Executive Agency (Chafea) acting under the mandate of the European Commission. The content of this article represents the views of the contractor (the EPHORT consortium) and is its sole responsibility; it can in no way be taken to reflect the views of the European Commission and/or Chafea or any other body of the European Union. The full HEALIT4EU report is available via https://ec.europa.eu/health/sites/health/files/health_policies/docs/2015_health_literacy_en.pdf.

### Search methods for identification of studies

Studies were identified by searching Medline, PubMed, EMBASE, CINAHL, the Cochrane library, PsychINFO, ERIC, Web of Science and SCOPUS. English, French and German language publications on health literacy intervention studies in EU countries. According to our knowledge, no research on health literacy has been done before 1995, therefore studies from January 1995 to Augusts 2018 were included. Recent reviews on health literacy that developed search strategies based on a list of key words and text words for use in the different databases were used to construct our own search strategy. For the search strategy, the reviews of Berkman et al. [13] and Dennis et al. [14] were combined into a new search strategy (see Additional file 1). Contrary to most other search strategies, this search strategy for health literacy publications explicitly included ‘functional health literacy’, ‘interactive health literacy’ and ‘critical health literacy’ [3]. As in Berkman et al. [13] we also used the terms ‘literacy’, ‘literate’, ‘reading skills’, ‘writing skills’ etc.). This led to 2515 publications in PubMed and Embase alone. All these abstracts were screened by two researchers but it turned out that the majority of these publications did not address health literacy (or health related issues) at all. Therefore we limited the search terms for health literacy by leaving out the terms of general literacy, reading and writing skills and dyslexia. The terms used in our search strategies to find ‘interventions studies’ were the same search terms as used by Dennis et al. [14].

### Types of studies

All studies that describe an intervention study with one of the following designs were included: randomized controlled trials, quasi randomized controlled trials, controlled before and after studies or interrupted time series. Studies with no original data, studies with only case report and studies with only ecological data were excluded.

Studies involving people living in one or more member states of the EU were included. The Member States of the EU are Austria, Belgium, Bulgaria, Croatia, Cyprus, Czech Republic, Denmark, Estonia, Finland, France, Germany, Greece, Hungary, Ireland, Italy, Latvia, Lithuania, Luxembourg, Malta, Netherlands, Poland, Portugal, Romania, Slovakia, Slovenia, Spain, Sweden and the United Kingdom.

### Types of interventions

Studies with an intervention that focused on health literacy were included. Interventions at population level, as well as interventions on specific populations and individual level were searched for and included. Studies on the basic experimental science of reading ability were excluded as were studies examining normal reading development in children and studies about dyslexia. Contrary to the strategy of Berkman et al. [11], the search was not limited to publications mentioning the use of a health literacy measurement tool, because the way health literacy in Europe was measured differed from the way described in reviews with predominantly American studies (where validated health literacy instruments are used more often).

### Types of outcome measures

Studies that described an outcome measure related to health literacy were included. These outcome measure included among others: knowledge, skills, attitudes, self-efficacy, stages of change, motivation and patient activation, behaviour change, health care access, service use, health status, costs of care.

### Data collection and analysis

The study selection consisted of two phases: first the selection on title and/or abstract and second the selection of the remaining articles based on full text. The search results were screened by two researchers each in two independent phases (JH, MH (1995–2014); BV, BS (2015–2018)). Consensus meetings were held with the researchers of both phases to resolve disagreements. A 20% sample of the excluded scientific publications was screened by a third researcher (JR 1995–2018). Studies were included if they met the inclusion criteria.

The abstracts were systematically screened on the basis of our in- and exclusion criteria. In case a publication did not meet a criterion, the publication was excluded and the next publication was screened. Of all the studies, fulfilling the inclusion criteria, full texts were read. For the inclusion of full texts the same in- and exclusion criteria were used. To assess the quality of the studies the “Quality Assessment Tool For Quantitative Studies” developed by the Effective Public Health Practice Project (EPHPP) [16] was used. The criteria for quality in this tool include selection bias, study design, confounders, blinding, data collection methods, withdrawals, intervention integrity and data analysis. The *gl*obal rating is calculated using information across all six domains (selection bias, study design, confounders, blinding, data collection methods & withdrawals and drop-outs): strong (no weak ratings), moderate (one weak rating), or weak (two or more weak ratings)*.*

## Results

**Fig. 1:**
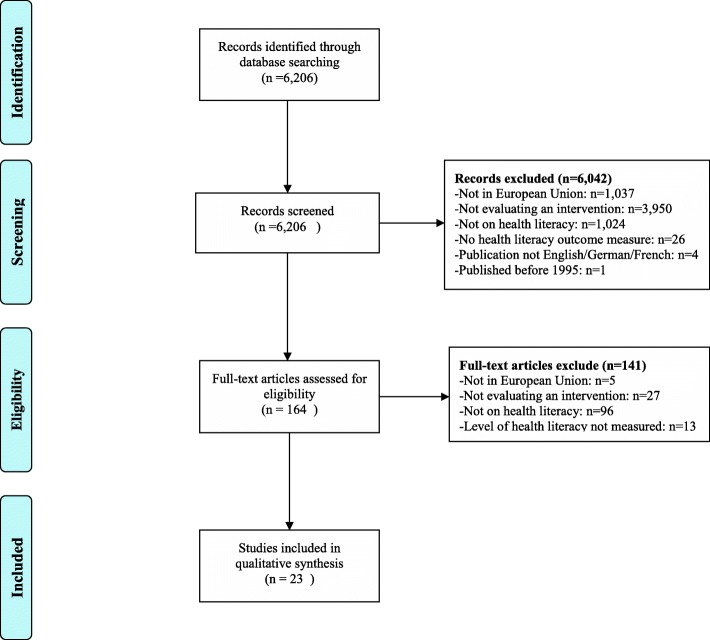
PRISMA diagram

Our literature search yielded 6206 publications between 1995 and mid-2018. Of these publications 6042 (97%) were excluded based on title and abstract because they did not fulfil one or more of the inclusion criteria: 3950 (65%) were excluded because not describing an intervention, 1037 (17%) did not meet the first criterion (being conducted in one or more of the European Member States), 1024 (17%) of the European interventions were excluded because they were not focusing on health literacy and 26 (1%) of the studies were excluded because there was no health literacy outcome measure (see also Fig. 1, PRISMA diagram). The remaining 164 publications were retrieved in full text for further assessment, of which 141 failed to meet the inclusion criteria. The main reason for excluding full texts was that they were unrelated to health literacy. Finally, 23 articles were included.

### Principal findings

There were not a sufficient number of studies with similar outcome measures or similar interventions to consider quantitative analysis (meta-analysis or statistical pooling) of data; therefore a qualitative analysis was performed. The 23 included intervention studies and their characteristics are summarized in Table 1. The references, the evidence tables, the intervention type and outcome of each of these studies can be found in Table 2. All studies were conducted in North-western Europe, no studies from Eastern and Southern European countries were found. All studies except one [17] were interventions developed for adults.

**Table 1:** Characteristics of interventions included (*n* = 23)

Characteristics	
Publication year	2005 (*n* = 2)
	2007 (*n* = 1)
	2010 (*n* = 2)
	2011 (*n* = 3)
	2012 (*n* = 5)
	2013 (*n* = 3)
	2015 (*n* = 2)
	2016 (*n* = 3)
	2017 (*n* = 2)
Country	Austria (*n* = 3)^a^
	Denmark (*n* = 3)^a^
	Germany (*n* = 6)^a^
	Ireland (*n* = 2)^a^
	Netherlands (*n* = 4)
	United Kingdom (*n* = 8)^a^
	Taiwan (*n* = 1)^a^
Study design	Cohort analytic group design (*n* = 2)
	Cohort (one group pre/post) (*n* = 9)
	Controlled trial (*n* = 5)
	Interrupted time series (*n* = 1)
	Randomized controlled trial (RCT) (*n* = 5)
	Observational study (*n* = 1)
Study Setting	Community (*n* = 6)
	Health Professionals (*n* = 2)
	Outpatients (*n* = 5)
	Telephone and/or mail intervention (*n* = 10)
Duration of intervention and follow-up	No follow-up (*n* = 13)
	Follow up ≤3 months (*n* = 4)
	Follow-up> 3 months (*n* = 4)
	Unknown (*n* = 2)
Age of participants	Children (8–12) (*n* = 1)
	Adults (> 16) (*n* = 22)
Health Literacy Measure	Critical Health Competence List (*n* = 1)
	Brief questions to identify patient with inadequate health literacy (*n* = 1)
	Critical HL assessed by interview (*n* = 1)
	Level of Knowledge (*n* = 3)
	REALM-R (*n* = 2)
	Newest Vital Sign Test (*n* = 1)
	Level of reading ability (*n* = 2)
	Level of mild intellectual disabilities (*n* = 1)
	Numeracy competence (*n* = 4)
	Skills towards decision making in a health context (*n* = 1)
	Not specified (*n* = 1)
	Danish version of TOFHLA (*n* = 3)
	Dutch version of SAHL (*n* = 2)
Focus of included studies	Disease specific: (*n* = 9) of which Diabetes (*n* = 5), Cancer (*n* = 1), COPD (*n* = 3), Osteoarthritis (*n* = 1), Rheumatoid arthritis (*n* = 1), Multiple Sclerosis (*n* = 1), Renal patients (*n* = 1);
	People working or using health care (*n* = 2)
	Hard to reach groups (*n* = 3)
	Outpatients not specified (*n* = 1)
	People with mild intellectual disabilities (*n* = 1)
	Smokers (*n* = 1)
	General population (*n* = 4)
Health issues	Diabetes, Cancer, OA, MS, RA, participation in treatment, knowledge, understanding of medication, adherence to medication, interpretation of information about treatment, appraisal skills in judging medical information, self-management, active participation in treatment, empowerment, self-management skills and confidence, motivation to self-manage, risk-communication, decision-making in medical treatment, symptom monitoring and recognition, reaching disadvantaged groups, health promotion, health status, social participation and integration, access to health care, health care use, communication of medical information.

^a^The total number of countries is more than 23, because the study of Muller et al. was performed in the United Kingdom, Austria, Germany, Ireland, and Taiwan

**Table 2:** Quality assessment (using the EPHPP), intervention type and outcome of reviewed studies *N* = 23

Study (primary author)	Selection bias	Study design	Confounders	Blinding	Data collection methods	Withdrawals and drop-outs	Global rating	intervention type	Dimension of health literacy	Outcome
Meppelink et al., 2015 [24]	S	S	S	M	S	S	Strong	Web-based intervention	Functional	Functional health literacy
									Critical	Critical health literacy
Elbert et al., 2016 [26]	M	S	S	M	S	W	Moderate	Web-based intervention	Functional	Healthy lifestyle
Gilbert et al., 2012 [27]	M	S	S	S	W	M	Moderate	Tailored information leaflet	Functional	Healthy lifestyle
									Critical	
Meppelink et al., 2015 [25]	W	S	S	M	S	M	Moderate	Web-based intervention	Functional	Functional health literacy
									Critical	Critical health literacy
Heasum et al., 2017 [23]	M	S	S	W	S	M	Moderate	Web-based intervention	Functional	Functional health literacy
Sahm et al., 2011 [18]	M	S	S	M	M	W	Moderate	Tailored information leaflet	Functional	Understanding of drug label
Walker et al., 2007 [19]	M	S	S	M	S	W	Moderate	Tailored information leaflet	Functional	Knowledge
Webb et al., 2008 [32]	M	M	W	M	M	M	Moderate	Group-based intervention	Functional	Self-management
									Interactive	Healthy lifestyle
										Health status
										Access to health care
										Ability to make treatment choices
										Symptom recognition and monitoring
Berger et al., 2013 [29]	W	M	W	M	W	M	Weak	Group-based intervention	Critical	Level of critical health literacy
										Self-management
Blanson Henkeman et al., 2013 [17]	W	S	W	W	S	S	Weak	Individual, personal contact	Functional	Knowledge
										Self-management
Boxell et al., 2012 [20]	W	M	W	M	S	S	Weak	Tailored information leaflet	Functional	Symptom recognition and monitoring
Garcia-Retamero et al., 2010 [35]	W	M	S	M	M	W	Weak	Web-based intervention	Functional	Understanding of health risk
Galesic et al., 2011 [36]	W	S	W	M	M	W	Weak	Aids to support numerical concepts	Functional	Understanding of health risk
Galesic et al., 2013 [37]	W	S	M	W	W	W	Weak	Aids to support numerical concepts	Functional	Ability to make treatment choices
Haesum et al., 2016 [21]	W	M	W	M	M	S	Weak	Web-based intervention	Functional	Functional health literacy
										Self-management
										Shared decision making
Kasper et al., 2005 [38]	M	M	M	W	M	W	Weak	Aids to support numerical concepts	Functional	Level of functional health literacy
										Shared decision making
Lilholt et al., 2016 [22]	M	M	W	M	W	M	Weak	Web-based intervention	Functional	Functional health literacy
										Health status
										Self-management
										Shared decision making
Long et al., 2011 [30]	W	M	W	M	W	W	Weak	Self-management support by call-center	Functional	Knowledge
									Critical	Empowerment
										Self-management
										Critical health literacy
Matic-Strametz et al., 2012 [33]	W	S	W	W	M	S	Weak	Group-based intervention	Functional	Knowledge
									Critical	Critical health literacy
Muller et al., 2017 [34]	W	S	S	M	S	W	Weak	Web-based intervention	Functional	Functional health ltieray
										Healthy lifestyle
										Self-management
Neville et al., 2005 [28]	W	M	W	M	W	W	Weak	Web-based intervention	Functional	Understanding of health risk
								Multi-component programme		Self-management
								Individual, personal contact		Understanding of drugs label
Reiter et al., 2012 [39]	W	M	W	W	W	W	Weak	Group-based intervention	Functional	Knowledge
								Multi-component programme		Healthy lifestyle
										Health status
White et al., 2012 [31]	S	S	W	W	S	W	Weak	Individual, personal contact	Functional	Self-management
									Critical	Ability to make treatment choices
										Access to health care
										Empowerment
										Healthy lifestyle

*S* strong, *M* moderate*, W* weak*.* Global Rating is calculated using information across all six domains: strong (no weak ratings), moderate (one weak rating), or weak (two or more weak ratings)

### Health literacy measure

Studies varied considerably in their measurement of health literacy. Commonly used instruments in the USA to assess health literacy such as the Raped Estimate of Adult Literacy in Medicine (REALM) [18, 19], the Newest Vital Sign (NVS) [20], Test Of Functional Health Literacy in Adults (TOFHLA) [21–23], and the Short Assessment of Health Literacy (SAHL) [23, 24] were used in eight studies. All these measures focus on functional health literacy. Four other studies also focused on functional health literacy skills by assessing reading ability [25, 26] or the level of mild intellectual disability [27]. Three studies measured critical health literacy skills by questionnaire [28] or interview [29] or assessing skills towards decision-making [30]. The study by Webb et al. [31] focussed on functional and interactive health literacy skills by measuring health literacy as the level of verbal and cognitive abilities. Three studies measured health literacy by the level of disease-specific knowledge [20, 32, 33]. One study measured health literacy by the Brief questions to identify inadequate health literacy [34]. In one study the way health literacy was measured was not specified [31]. Numeracy was assessed in four studies [35–38].

### Type of intervention

There was also a huge variation in the type of interventions given: group interventions, individual interventions, web-based interventions, one component interventions (e.g. an information leaflet) and multi-component interventions including chat-groups, lectures, training sessions, a help-desk, computer programs and leaflets among others. Most interventions were web-based interventions (*n* = 9). The web-based interventions were conducted during the most recent years, most of them (*n* = 7) in 2015, 2016 and 2017. In only one study it was explicitly mentioned that the patients were involved in the development of the intervention on a module designed for the development of a decision aid about MS-immunotherapy [38].

### Study design

Five studies used a Randomised Clinical Trial (RCT) design [19, 23, 26, 27, 34] and five studies a Controlled Clinical Trial (CCT) design [18, 21, 24, 25, 33]. In two studies two groups were compared pre- and post-test (Cohort analytic design) [17, 28], but most studies (*n* = 9) used the same group that was pretested and post-tested immediately after the intervention (Cohort study). The study by White et al. [31] used an interrupted time series design and there was one observational study. The type of design in combination with the frequent missing or nor reported use of covariates makes that for most studies (*n* = 15) the quality was rated as weak (EPHPP [16], Table 2). The quality of seven studies was rated as moderate on the base of the EPHPP [16] assessment tool and one study was judged as strong.

### Types of intervention studies

The types of interventions in the 23 studies could be categorized as follows;

1. Interventions aimed at improving (aspects of) the health literacy level of individuals.
2. Interventions that were specifically tailored to different health literacy levels.
3. General interventions that aimed at improving health outcomes, which described the specific effects for patients with different health literacy or numeracy levels.

#### Interventions aimed at improving (aspects of) health literacy

A group training of 2 × 2,5 days in evidence based-medicine for patients, patient counsellors, consumer representatives and healthcare professionals resulted in a significant increase in health related knowledge and in the level of critical health literacy of the participants [29]. In the evaluation of the training they stated that they had broadened their knowledge, were more critical in handling health information and considered themselves more confident on making the right decisions on the basis of the information they found. The content of the training was tailored to the needs of the participants. A second group intervention [39] specifically focused on so-called ‘hard to reach’ groups (e.g. unemployed women of minority groups and female migrants from Islamic backgrounds). This intervention combined different elements: computer courses, lectures, and language training. Topics related to health and well-being were being discussed. Also this intervention led to an increase in knowledge and comprehensive health literacy. Another group intervention targeted patients with mild intellectual disabilities and was tailored to their verbal and cognitive abilities. In the training, patients were taught how and when to access healthcare [22]. The evaluation showed that the intervention had a significant positive effect on the participants’ ability to recognize disease symptoms, identify illnesses and choose appropriate courses for action.

An intervention that was developed to improve self-care among diabetes patients was evaluated after two years. The patients had received tailored tele-carer education as well as support to change specific lifestyle behaviours [30]. The evaluation showed that these diabetes patients were better able to use knowledge in their day-to-day self-care and expressed a greater control over their self-care decision-making. A UK community study that evaluated the impact of a self-care skills training initially (after 6 months) found a positive effect on decision making skills regarding use of health services (critical health literacy). However, after 12 months the effect was no longer found [31]. In three studies in Denmark, the tele-homecare intervention 'Telekit' was evaluated. The Telekit focuses on the management of COPD in general, how to manage COPD during exacerbations and collect date on the current state of the patient’s health. Both studies did not found a significant difference on functional health literacy [21–23]. The Telekit increases the feeling of insecurity, greater freedom, more control and greater awareness of symptoms [22].

Five interventions specifically focused on the improvement of numeracy skills, i.e. the ability to understand numerical risk information [18, 21, 35–37]. The evaluations of these interventions had similar conclusions. In general numerical information is presented in ways too difficult for people with low competencies. Another way of presenting (e.g. by using visual aids and/or lowering the level of detail of information) led to improved understanding in participants with low numeracy competencies.

#### Interventions tailored to different health literacy levels

Three studies [17, 27, 28] performed an evaluation with an intervention and a control group, comparing the outcome variables. In one study among children with diabetes (age 8–12) the impact of a personalized robot on diabetes knowledge and motivation for self-management was compared to a neutral robot. The reactions of the personalized robot were adjusted to the knowledge level of the child. In the evaluation, children in the intervention group (with the personalized robot) scored higher on diabetes knowledge and motivation for self-management. A tailored training programme on peritoneal dialysis for renal patients with low health literacy resulted in lower incidence of peritonitis and stronger feelings of control and ownership over treatment among the participants in the intervention group, as well as less supervision time needed of nurses [28]. The intervention comprised lowering the amount of written information and using more verbal material, and reducing the use of medical jargon. A computer-tailored intervention for smoking-cessation (booklet and web-based programme) was compared to a general self-help booklet. The tailored approach led to more attempts to quit smoking as well as higher abstinence rates, specifically for participants with lower literacy levels [27]. An intervention that was tailored to the verbal and cognitive abilities of patients with mild intellectual disabilities was evaluated in a one group pre/post-test design [32]. The evaluation showed there was an improvement in symptom recognition, better health-related decision making, improved understanding of medical procedures and a better ability in formulating personal health goals.

Three studies focused on the way of presenting information to persons with different health literacy levels [24, 25, 34]. One study varied in presenting information on spoken versus written text and illustration versus animation. In almost all conditions, the high health literate persons had a better recall on information compared to the low health literate persons, except for the spoken animations. In the spoken animation condition, the low health literate persons recalled the same amount of information as the high literate persons. The other study varied in presenting information on illustrated versus text-only and in not difficult versus difficult texts. Persons with low and high health literacy recalled the not difficult information better than difficult information. Illustrated text improved the recall and attitudes in low health literate persons and had no effect in high health literate persons. Another study stated audio-visual leads to better knowledge. The study also stated that clear, person-based intervention development is more important than interactivity and audio-visual presentation to improve health literacy outcomes.

#### General interventions that aimed at improving health outcomes, which described the specific effects for patients with different health literacy or numeracy levels

In general, patients with low health literacy benefit less from general interventions compared to patients with higher levels of literacy, e.g. with respect to understanding medication labels [18, 35] and other health messages [19, 20]. In a study on the knowledge level of rheumatoid arthritis patients after being exposed to a pictorial ‘mind map’ together with a arthritis campaign booklet, analysis showed that less literate participants gained fewer knowledge from both the booklet alone and the booklet in combination with the mind map, compared to high literate patients [19]. Similarly, a leaflet was developed to improve gynaecological cancer symptom awareness and to reduce barriers to access medical services [20]. Though in general after reading the leaflet awareness improved and barriers to access medical services were reduced, these effects were less in patients with lower health literacy. In general, patients with low levels of health literacy were found to experience more barriers to access health care services

Four studies reported on outcomes relevant for the daily management of chronic illness or health in general such as knowledge, empowerment, ability to self-manage, decision-making skills, ability to taken an active role in treatment. Increased levels of health literacy were associated with higher levels of empowerment, better decision-making skills, and a more active role in treatment [29–32]. The evidence were graded as weak due to the fact that results mainly came from uncontrolled studies and results were often based on small groups or a limited number of observations. One study focused on a mobile phone app intervention targeting fruit and vegetable consumption. The information provided via the app where either textual or auditory tailored to the person’s characteristics. The app increased the fruit and vegetable intake, but only in persons with high health literacy [26].

## Discussion

In this systematic review the evidence on the effectiveness of health literacy interventions in the EU published from 1995 until mid-2018 was assessed. There were not a sufficient number of studies with similar outcome measures or similar interventions to consider quantitative analysis (meta-analysis or statistical pooling) of data; therefore a qualitative analysis was performed. The evidence collected gives insight into the gaps in research in the context of the European Union, compared to the evidence presented in the already published reviews outside Europe, and provide recommendations for research. To our knowledge, this is the first systematic review on health literacy interventions in the EU context. The results of this review are compared to the results of reviews in the USA context.

In total, 23 intervention studies were identified. The interventions described in these studies either (a) aimed at improving (aspects of) health literacy, (b) were specifically tailored to different health literacy levels or (c) were general interventions that aimed at improving health outcomes, which described the specific effects for patients with different health literacy or numeracy levels. As was found in other review studies [13, 14, 40] most interventions focus on functional health literacy, fewer (also) target interactive or critical health literacy.

The studies varied with respect to their study design, measurement instruments and outcomes. Health literacy was also operationalized and measured differently, thus impeding comparability of the results. Most studies did not give information whether their study results were stratified across health literacy levels. This was also concluded in the review of D ‘Eath et al. [40]. As a result of this, it is not possible to measure the impact of interventions on people with varying levels of health literacy. The quality of most studies was weak (15) or moderate (7). Only one was rated as strong. The number of RCT’s or controlled studies was limited.

Because of the low quality of the studies no firm conclusions can be drawn with respect to the effective components of health literacy interventions. It seems that the type of intervention (e.g. group, individual, community based) is not of major importance. However, three factors are likely to be distinctive of promising interventions: (1) they tailor their activities to the needs of the (low health literate) participants, (2) they also address interactive and/or critical skills (instead of knowledge only) and (3) they present the information in an appropriate way, i.e. not difficult and using animated spoken text. Studies that also focus on interactive and/or critical skills led to improvements in outcomes such as motivation, knowledge, empowerment and self-confidence. These findings are congruent with those from the review by Berkman et al. [13]. Interventions that present the information in an appropriate way results in better recall, positive attitudes and more informed decision making [24, 25, 34].

## Conclusions

Despite the small number of studies, findings from the EU are in line with the results from other international reviews [6–8, 13, 14, 40]. Most interventions in this review focus on functional level of health literacy, these results were also found in the other USA reviews [6, 7, 13, 14, 40]. In our review we found that not all studies identified whether the study results were stratified across health literacy levels. These results were also found by the USA review [40]. Similar to the review of Berkman et al. [13] we identified that increased levels of health literacy were associated with higher levels of empowerment, better decision making skills, and a more active role in treatment. In our review, the quality of most of the studies were rated as low. In contrast to our included studies, the quality of most of the included studies of the USA reviews [6–8, 13, 14, 40] were rated as moderate/fair and high/good.

There are definitely considerable gaps in the research evidence concerning which interventions are most effective in improving health literacy or health literacy related outcomes in Europe. In order to be able to draw firm conclusions, there should be more agreement among researchers about the definition of health literacy, and more systematic use of validated measurement tools in interventions as a “golden standard”. In the past years several studies on the development, translation and validation of (both subjective and objective) health literacy measurement instruments have been done. As a consequence, the assessment of health literacy varies depending on the setting and scope of the health literacy definition. The results of future intervention research then become more comparable and generalizable, leading to a more rapid insight in what constitutes effective health literacy interventions in the EU context.

New developed interventions should be tailored to the needs of the patients; address functional, critical and interactive skills and the way of presenting should be not difficult animated spoken text. Web-based interventions might be suitable for patients that have digital skills, but also blended interventions (combining face-to-face with online activities) and other types of interventions might integrate these three factors in their design. Future research should focus on the assessment of such interventions and use stronger designs e.g. in well-reported, large-sampled randomized controlled trials.

## Additional file

Additional file 1: Final search strategy (DOCX 13 kb)

## Abbreviations

EPHPP: Effective Public Health Practice Project
EU: European Union
HLS-EU: European Health Literacy Project
PRISMA: Preferred Reporting Items for Systematic Reviews and Meta Analyses
USA: United States of America

## References

[1] European Commission: Together for health: a strategic approach for the EU 2008-2013. Com (2007) 630 final. 2007.

[2] Sorensen, K., Van den Broucke, S., Fullam, J., Doyle, G., Pelikan, J., Slonska, Z., & Brand H. (HLS-EU) Consortium Health Literacy Project European. (2012). Health literacy and public health: A systematic review and integration of definitions and models. BMC Public Health, 12, 80–2458-12-80. doi:10.1186/1471-2458-12-80 . 10.1186/1471-2458-12-80 PMC3292515 22276600

[3] Nutbeam D (2000). Health literacy as a public goal: a challenge for contemporary health education and communication strategies into the 21st century. Health Promot Int.

[4] Rasu RS, Bawa WA, Suminski R, Snella K, Warady B (2015). Health literacy impact on national healthcare utilization and expenditure. Int J Health Policy Manag.

[5] WHO Commission on the Social Determinants of Health (2007). Achieving health equity: from root causes to fair outcomes.

[6] DeWalt DA, Hink A (2009). Health literacy and child health outcomes: a systematic review of the literature. Pediatrics.

[7] Mantwill S, Monestel-Umana S, Schulz PJ (2015). The relationship between health literacy and health disparities: a systematic review. PLoS One.

[8] Sanders LM, Federico S, Klass P, Abrams MA, Dreyer B (2009). Literacy and child health: a systematic review. Archives of Pediatrics & Adolescent Medicine.

[9] Nutbeam D (2008). The evolving concept of health literacy. Soc Sci Med.

[10] Rice T, Rosenau P, Unruh LY, Barnes AJ, Saltman RB, van Ginneken E (2013). United States of America: health system review. Health Syst Transit.

[11] Loewenson R, Simpson S (2017). Strengthening integrated care through population-focused primary care services: international experiences outside the United States. Annu Rev Public Health.

[12] Sheridan SL, Halpern DJ, Viera AJ, Berkman ND, Donahue KE, Crotty K (2011). Interventions for individuals with low health literacy: a systematic review. J Health Commun.

[13] Berkman ND, Sheridan SL, Donahue KE, Halpern DJ, Viera A, Crotty K, et al. Health literacy interventions and outcomes: An updated systematic review. Evid Rep Technol Assess. 2011;(199):1–941. PMC4781058 23126607

[14] Dennis S, Williams A, Taggart J, Newall A, Denney-Wilson E, Zwar N, Harris MF (2012). Which providers can bridge the health literacy gap in lifestyle risk factor modification education: A systematic review and narrative synthesis. BMC Fam Pract.

[15] Moher D, Liberati A, Tetzlaff J, Altman DG, PRISMA Group (2010). Preferred reporting items for systematic reviews and meta-analyses: The PRISMA statement. Int J Surgery.

[16] Effective Public Health Practice Project (EPHPP). (2009). Quality assessment tool for quantitative studies. https://www.nccmt.ca/knowledge-repositories/search/14. Accessed 23 Mar 2017.

[17] Blanson Henkemans OA, Bierman BP, Janssen J, Neerincx MA, Looije R, van der Bosch H, van der Giessen JA (2013). Using a robot to personalise health education for children with diabetes type 1: a pilot study. Patient Educ Couns.

[18] Sahm LJ, Wolf MS, Curtis LM, Behan R, Brennan M, Gallwey H, Mc Carthy S (2012). What's in a label? An exploratory study of patient-centered drug instructions. Eur J Clin Pharmacol.

[19] Walker D, Adebajo A, Heslop P, Hill J, Firth J, Bishop P, Helliwell PS (2007). Patient education in rheumatoid arthritis: the effectiveness of the ARC booklet and the mind map. Rheumatology (Oxford).

[20] Boxell EM, Smith SG, Morris M, Kummer S, Rowlands G, Waller J (2012). Increasing awareness of gynecological cancer symptoms and reducing barriers to medical help seeking: does health literacy play a role?. J Health Commun.

[21] Korsbakke Emtekaer Haesum L, Ehlers L, Hejlesen OK (2016). Interaction between functional health literacy and telehomecare: Short-term effects from a randomized trial. Nurs Health Sci.

[22] Lilholt PH, Haesum LK, Ehlers LH, Hejlesen OK (2016). Specific technological communication skills and functional health literacy have no influence on self-reported benefits from enrollment in the TeleCare north trial. Int J Med Inform.

[23] Haesum LKE, Ehlers LH, Hejlesen OK (2017). The long-term effects of using telehomecare technology on functional health literacy: results from a randomized trial. Public Health.

[24] Meppelink CS, Smit EG, Buurman BM, van Weert JC (2015). Should we be afraid of simple messages? The effects of text difficulty and illustrations in people with low or high health literacy. Health Commun.

[25] Meppelink CS, van Weert JC, Haven CJ, Smit EG (2015). The effectiveness of health animations in audiences with different health literacy levels: an experimental study. J Med Internet Res.

[26] Elbert SP, Dijkstra A, Oenema A (2016). A mobile phone app intervention targeting fruit and vegetable consumption: the efficacy of textual and auditory tailored health information tested in a randomized controlled trial. J Med Internet Res.

[27] Gilbert HM, Leurent B, Sutton S, Alexis-Garsee C, Morris RW, Nazareth I (2013). ESCAPE: a randomised controlled trial of computer-tailored smoking cessation advice in primary care. Addiction.

[28] Neville A, Jenkins J, Williams JD, Craig KJ (2005). Peritoneal dialysis training: a multisensory approach. Perit Dial Int.

[29] Berger B, Gerlach A, Groth S, Sladek U, Ebner K, Muhlhauser I, Steckelberg A (2013). Competence training in evidence-based medicine for patients, patient counsellors, consumer representatives and health care professionals in Austria: a feasibility study. Zeitschrift Fur Evidenz, Fortbildung Und Qualitat Im Gesundheitswesen.

[30] Long AF, Gambling T (2012). Enhancing health literacy and behavioural change within a tele-care education and support intervention for people with type 2 diabetes. Health Expect.

[31] White A, South J, Bagnall AM, Forshaw M, Spoor C, Marchant P, Witty K (2012). The self-care for people initiative: the outcome evaluation. Prim Health Care Res Dev.

[32] Webb J, Stanton M (2009). Better access to primary healthcare for adults with learning disabilities: evaluation of a group programme to improve knowledge and skills. Br J Learn Disabil.

[33] Matic-Strametz M, Strametz R, Bohrt K, Ochsendorf F, Weberschock T (2013). Students in training to become biology teachers - a controlled phase II trial (NCT01567267). [Evaluation eines Lehrkonzepts in EbM fur Lehramtsstudierende der Biologie - eine kontrollierte Phase-II-Studie (NCT01567267)]. Zeitschrift Fur Evidenz, Fortbildung Und Qualitat Im Gesundheitswesen.

[34] Muller I, Rowsell A, Stuart B, Hayter V, Little P, Ganahl K (2017). Effects on engagement and health literacy outcomes of web-based materials promoting physical activity in people with diabetes: an international randomized trial. J Med Internet Res.

[35] Garcia-Retamero R, Galesic M (2010). Who profits from visual aids: overcoming challenges in people's understanding of risks [corrected]. Soc Sci Med.

[36] Galesic M, Garcia-Retamero R (2011). Communicating consequences of risky behaviors: life expectancy versus risk of disease. Patient Educ Couns.

[37] Galesic M, Garcia-Retamero R (2013). Using analogies to communicate information about health risks. Appl Cognit Psychol.

[38] Kasper J, Kopke S, Muhlhauser I, Heesen C (2006). Evidence-based patient information about treatment of multiple sclerosis--a phase one study on comprehension and emotional responses. Patient Educ Couns.

[39] Reiter, A. (2012). “Evaluierung “Gesundheit kommt nachhause – Mehr Wissen hilft!”.”.

[40] D’Eath M, Barry MM, Sixsmith J (2012). Rapid evidence reviews of interventions for improving health literacy.

