# Supplementary material for: Evidence on the effectiveness of health literacy interventions in the EU: a systematic review
Source: BMC Public Health. 2018 Dec 29;18:1414. doi: 10.1186/s12889-018-6331-7 (PMC6310940; doi:10.1186/s12889-018-6331-7)
Supplement: Supplementary file 1 — Final search strategy (DOCX 13 kb) [file 12889_2018_6331_MOESM1_ESM.docx]

**Annex A**

*Final search strategy*

Pubmed Search Health Literacy and interventions

("Health Literacy"[Mesh] OR "Health Literacy"[tiab] OR "health knowledge"[tiab] OR "medical data interpretation"[tiab] OR "health competence"[tiab]) OR (("Health"[Mesh] OR health[tiab] OR patient*[tiab]) AND (literacy[tiab] OR literate[Tiab] OR "reading skills"[tiab] OR "reading ability"[tiab] OR "reading level"[tiab] OR "writing level"[tiab] OR "writing ability"[tiab] OR "writing skills"[tiab] OR numeracy[tiab] OR analphabetism[tiab])) AND ("health promotion"[tiab] OR programme[tiab] OR "Evaluation Studies"[Publication Type] OR evaluation[tiab] OR "Evaluation Studies as Topic"[Mesh] OR "Health Promotion"[Mesh] OR "Government Programmes"[Mesh] OR "Intervention Studies"[Mesh] OR intervention[tiab] OR effect*[tiab] OR "health communication"[mesh] OR "Health Communication"[tiab] OR policy[tiab] OR "patient education as topic"[mesh] OR "patient education"[tiab]) AND ("Europe"[Mesh] OR "European union"[mesh] OR Europe[tiab] OR European[tiab] OR "eu countries" OR Austria[tiab] OR Austrian*[tiab] OR Belgium[tiab] OR Belgian*[tiab] OR bulgaria[tiab] OR Bulgarian*[tiab] OR Croatia[tiab] OR Croatian*[tiab] OR Cyprus[tiab] OR Cypriots[tiab] OR Czech*[tiab] OR Denmark[tiab] OR Danish[tiab] OR danes[tiab] OR Estonia[tiab] OR Estonian*[tiab] OR finland[tiab] OR finnish[tiab] OR finns[tiab] OR france[tiab] OR French[tiab] OR Germany[tiab] OR german*[tiab] OR Greece[tiab] OR greek*[tiab] OR hungary[tiab] OR Hungarian*[tiab] OR Ireland[tiab] OR irish[tiab] OR Italy[tiab] OR Italian*[tiab] OR Latvia[tiab] OR Latvian*[tiab] OR Lithuania[tiab] OR Lithuanian*[tiab] OR Luxembourg[tiab] OR Luxembourg*[tiab] OR malta[tiab] OR maltese[tiab] OR Netherlands[tiab] OR dutch[tiab] OR Poland[tiab] OR polish[tiab] OR poles[tiab] OR Portugal[tiab] OR portuguese[tiab] OR Romania[tiab] OR Romanian*[tiab] OR Slovakia[tiab] OR Slovaks[tiab] OR Slovenia[tiab] OR Slovene*[tiab] OR spain[tiab] OR Spanish[tiab] OR Spaniards[tiab] OR Sweden[tiab] OR Swedish[tiab] OR swedes[tiab] OR "united kingdom"[tiab] OR "England"[tiab] OR "britain"[tiab] OR british[tiab] OR Austria[ad] OR belgium[ad] OR bulgaria[ad] OR croatia[ad] OR cyprus[ad] OR czech[ad] OR denmark[ad] OR estonia[ad] OR finland[ad] OR france[ad] OR germany[ad] OR greece[ad] OR hungary[ad] OR ireland[ad] OR italy[ad] OR latvia[ad] OR lithuania[ad] OR luxembourg[ad] OR malta[ad] OR netherlands[ad] OR poland[ad] OR portugal[ad] OR romania[ad] OR slovakia[ad] OR slovenia[ad] OR spain[ad] OR sweden[ad] OR UK[ad]) NOT (editorial[pt] OR letter[pt] OR comment[pt] OR "Dyslexia"[Mesh] OR dyslectic[tiab] OR dyslexi*[tiab] OR Hispanic*[tiab] OR Mexican*[tiab] OR latina*[tiab] OR latino*[tiab]) AND hasabstract[text] AND ( "1995/01/01"[PDat] : "2018/08/31"[PDat]) AND (Dutch[language] OR German[language] OR French[language] OR English[Language])
